# Immunoproteomic identification and characterization of Ni^2+^-regulated proteins implicates Ni^2+^ in the induction of monocyte cell death

**DOI:** 10.1038/cddis.2017.112

**Published:** 2017-03-16

**Authors:** Annika Jakob, Franz Mussotter, Stefanie Ohnesorge, Lisa Dietz, Julian Pardo, Ian D Haidl, Hermann-Josef Thierse

**Affiliations:** 1 Laboratory for Immunology and Proteomics, Department of Dermatology and University Medical Center Mannheim, University of Heidelberg, Mannheim 68167, Germany; 2 German Federal Institute for Risk Assessment, Chemicals and Product Safety, Berlin 10589, Germany; 3 Department of Molecular Immunology, Biology III, University of Freiburg and Max-Planck-Institute for Immunobiology and Epigenetics, Freiburg 79108, Germany; 4 Functional Proteome Analysis, German Cancer Research Center (DKFZ), Heidelberg 69120, Germany; 5 Aragón I+D Foundation (ARAID), Zaragoza, Spain; 6 Department of Microbiology, Preventive Medicine and Public Health, University of Zaragoza/IIS Aragón, Zaragoza, Spain; 7 Biomedical Research Center of Aragón (CIBA), Aragón Health Research Institute (IIS Aragón), University of Zaragoza, Zaragoza, Spain; 8 Nanoscience Institute of Aragon (INA), University of Zaragoza, Zaragoza, Spain; 9 Dalhousie Inflammation Group, Department of Microbiology and Immunology, Dalhousie University, Halifax, NS, Canada

## Abstract

Nickel allergy is the most common cause of allergic reactions worldwide, with cutaneous and systemic effects potentially affecting multiple organs. Monocytes are precursors of not only macrophages but also dendritic cells, the most potent activators of nickel hypersensitivity. Monocytes are themselves important antigen-presenting cells, capable of nickel-specific T-cell activation *in vivo* and *in vitro*, in addition to being important for immediate innate immune inflammation. To elucidate early Ni^2+^-dependent inflammatory molecular mechanisms in human monocytes, a Ni^2+^-specific proteomic approach was applied. Quantitative two-dimensional (2D) differential gel electrophoresis and Delta2D software analyses coupled with matrix-assisted laser desorption/ionization mass spectrometry (MALDI-MS) revealed that Ni^2+^ significantly regulated 56 protein species, of which 36 were analyzed by MALDI-MS. Bioinformatics analyses of all identified proteins resulted in Ni^2+^-associated functional annotation clusters, such as cell death, metal ion binding, and cytoskeletal remodeling. The involvement of Ni^2+^ in the induction of monocyte cell death, but not T-cell death, was observed at Ni^2+^ concentrations at or above 250 *μ*M. Examination of caspase activity during Ni^2+^-mediated cell death revealed monocytic cell death independent of caspase-3 and -7 activity. However, confocal microscopy analysis demonstrated Ni^2+^-triggered cytoskeletal remodeling and nuclear condensation, characteristic of cellular apoptosis. Thus, Ni^2+^-specific peripheral blood mononuclear cell stimulation suggests monocytic cell death at Ni^2+^ concentrations at or above 250 *μ*M, and monocytic effects on immune regulation at lower Ni^2+^ concentrations.

The transition metal nickel (Ni^2+^) is the most common human contact allergen, triggering both innate and adaptive immune responses.^1, 2, 3, 4^ Potential sensitizing Ni^2+^ is not only used in medical devices like dental and surgical instruments, stents, and orthopedic implants but may also be released from jewelry, piercing materials, coins, mobile phones, and synthetic nanoparticles.^5, 6, 7^ During T-cell-mediated allergic contact dermatitis (ACD), these free Ni^2+^ ions interact directly with metal–complex-forming proteins to generate Ni^2+^-specific T-cell epitopes in human antigen-presenting cells, followed by activation of allergy-inducing naïve T cells (sensitization phase) or alternatively of Ni^2+^-tolerizing regulatory T cells (Treg).^8, 9, 10, 11, 12^ In a typical secondary hypersensitivity reaction re-exposure to Ni^2+^ results in recirculation of Ni^2+^-specific CD8^+^ Tc1/Tc17 and CD4^+^ Th1/Th17 T cells to the inflamed skin (elicitation phase) with all known clinical features of ACD.^3, 13, 14, 15^ Despite existing advances in characterizing Ni^2+^-specific human T-cell responses and functional Ni^2+^-interacting proteins, distinct molecular steps of Ni^2+^-specific inflammation remain to be elucidated.^16, 17, 18^

Research has also emerged demonstrating that Ni^2+^ and other ACD-inducing small reactive molecules (<500 Da) may have multifaceted effects on the immune system. Thus, it has been suggested that some contact allergens may – like some microbes – first induce innate irritancy and/or adjuvanticity stress signals before initiation of delayed-type adaptive immune reactions.^19, 20, 21, 22^ Depending on cell type and microenvironment studied, or chemical composition and concentrations applied, Ni^2+^-specific effects may include the generation of reactive oxygen species (ROS), Toll-like receptor 4 (TLR4) signal-transduction, Ca^2+^ channel blockade, danger molecule induction, immune cell differentiation, and cell death.^23, 24, 25, 26, 27^ In fact, it has been shown that Ni^2+^ is capable of modulating intracellular p38 mitogen-activated protein kinase (p38 MAPK) and NF-*κ*B pathways in monocyte-derived dendritic cells (DC), as well as IFN regulatory factor-1 and proinflammatory interleukin-12 (IL-12) production, a key element in Th1-driven immune responses.^28, 29, 30, 31, 32^ Similar Ni^2+^-dependent molecular effects have been observed in monocytes, demonstrating both Ni^2+^-regulated alteration of NF-*κ*B and p38 MAPK signaling.^33, 34^ Further studies suggest Ni^2+^-specific regulation of redox factor Nrf2, and Ni^2+^-dependent oxidative modification of cell-surface thiols, which has also been observed after incubation with the strong contact allergen 2,4-dinitrochlorobenzene.^34, 35, 36^

As monocytes are key players in the human system and are known precursors of phenotypically divergent DCs,^37, 38^ thereby affecting specific T-cell activation, we started to search for a more global proof of Ni^2+^-regulated proteins or Ni^2+^-specific pathways in primary human monocytes. Recent studies have shown that modern proteomics technologies, like 2D gel-based differential gel electrophoresis (DIGE) techniques and high-performance liquid chromatography with tandem mass spectrometric methods (LC/MS/MS), provide new qualitative and quantitative insights into molecular regulation of human monocytes and other immune cells.^9, 39, 40, 41^ Thus, to detect and identify proteins that are specifically regulated by Ni^2+^, and to evaluate potential allergen-related functional pathways in human CD14^+^ monocytes, a quantitative proteomic approach was established. Consecutive bioinformatics analysis of Ni^2+^-regulated proteins identified by MS revealed functional protein clusters being linked to distinct molecular processes including cell death. Hence, to examine potential apoptotic or toxic effects of human metal allergen Ni^2+^ in peripheral blood mononuclear cells (PBMCs) and monocytes, Ni^2+^-specific cell death was characterized.

## Results

### Isolation of human monocytes and quantitative detection of Ni^2+^-regulated proteins in human monocytes

**Figure 1:**
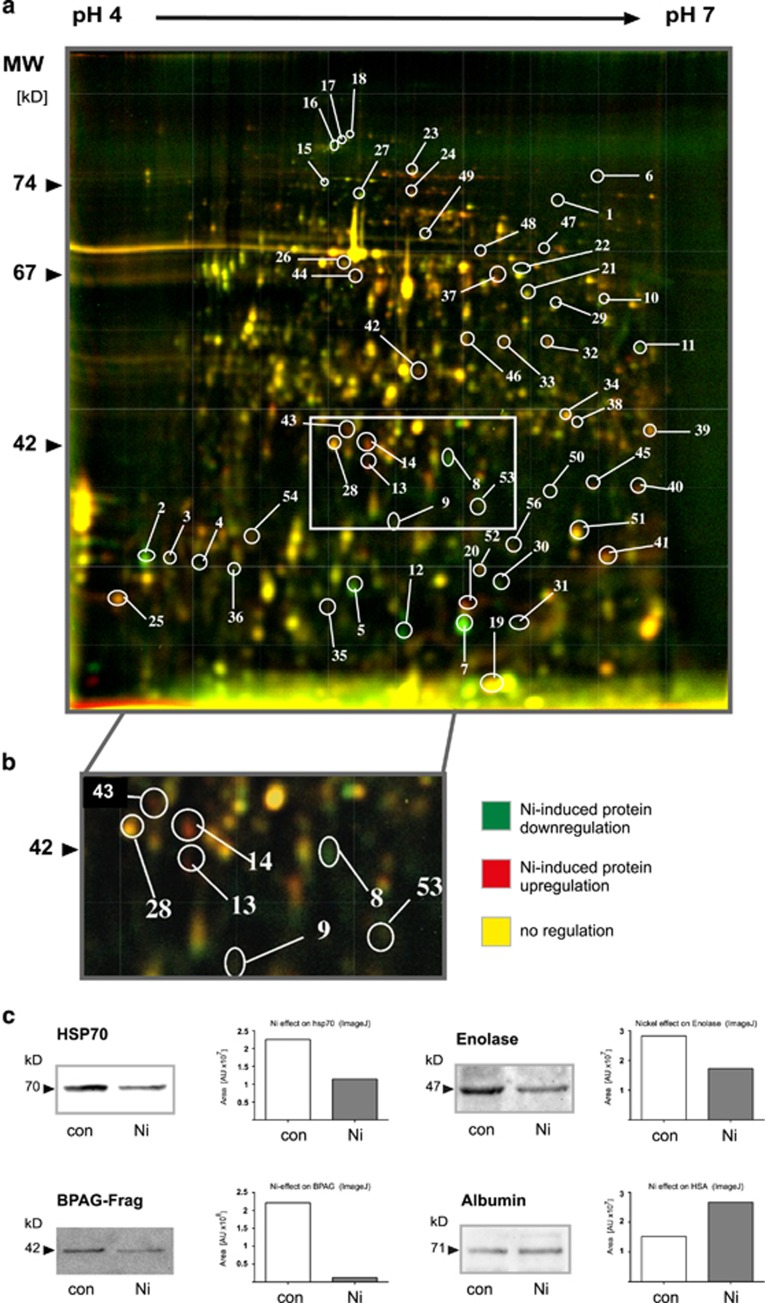
Differential protein profile of Ni^2+^-regulated proteins in human monocytes. CD14^+^-enriched monocytes were stimulated with 250 *μ*M NiSO_4_ and protein cell lysates of stimulated and unstimulated cells were labeled with CyDyes (DIGE approach) before being subjected to 2-DE (*n*=3–4). Scanned protein species pattern were analyzed by Delta2D software, and 56 significantly regulated protein spots were identified for subsequent MS analysis (**a**). Typical directly opposed Ni^2+^-regulated proteins are shown by enlargement (white rectangle), such as protein spot 8 (downregulated or lower concentrated; green), identified as bullous pemphigoid antigen 1, or protein spots 14 and 13 (upregulated or higher concentrated; red) identified as HSA protein (**b**). To confirm Ni^2+^ regulation as shown by DIGE and MS analysis, some complementary western blotting experiments were performed (**c**)

The purpose of our study was to investigate Ni^2+^-specific molecular effects on human CD14^+^ monocytes. MACS sorting of human PBMC provided >90% pure CD14^+^ monocytes, which were subsequently stimulated with increasing concentrations of Ni^2+^ (50–1000 *μ*M NiSO_4_). To detect and identify so far unknown Ni^2+^-regulated proteins in human monocytes, protein lysates from Ni^2+^-stimulated cells were applied to 2D-DIGE (Figure 1). Comparison of protein spot numbers significantly detectable on all 2D gels revealed an overall average of 532±126 protein spots per DIGE image. The number of proteins detected here appeared to be slightly higher than values calculated from silver-stained human B cells.^9^ A relatively high number of protein species, ~30.8±8.0% of the spots detected, were found to be regulated within the pH range selected (pH 4–7). The term protein species reflects possible detection of different isoforms or post-translational modifications of one protein, meaning that one protein may occur in different protein spots of just one electrophoretic run.^42, 43^ Stimulation of monocytes with 50 *μ*M NiSO_4_ demonstrated 29.7±9.6% totally regulated proteins, from which 4.3±3.1% were upregulated and 95.7±3.1% were downregulated protein spots, whereas incubation of cells with 250 *μ*M NiSO_4_ led to 32±5.8% regulated protein spots, with 9.2±3.6% upregulated and 90.8±9.6% downregulated proteins. Delta2D analyses of monocytes stimulated with 250 *μ*M NiSO_4_ revealed 56 significantly regulated protein species (Figure 1a), which were isolated from preparative gels for identification by MS analysis. Some exemplary opposed Ni^2+^-regulated proteins are shown by image magnification (Figure 1b), with spots 13 and 14 representing upregulated proteins (red) and protein spot 8 representing a downregulated protein (green) after cellular stimulation with 250 *μ*M NiSO_4_.

### Mass spectrometric identification, complementary confirmation of Ni^2+^-regulated proteins in human monocytes, and functional annotation clustering of candidate proteins

All significantly Ni^2+^-regulated proteins identified by Delta2D analyses were subjected to in-gel trypsinization before peptides were examined by matrix-assisted laser desorption/ionization mass spectrometry (MALDI-MS) measurement. MS spectra generated were used to identify each protein by matching to reference spectra of pre-existing protein sequence databases. In all, 39.3% of all excised proteins were successfully identified by this strategy, implying significant MASCOT scores based on protein-specific sequence coverage, peptide queries matched, and percentage peptide matches (Table 1). To confirm qualitatively and quantitatively Ni^2+^-dependent fluorescence-based DIGE and MALDI results, complementary western blotting experiments were performed. Four candidate proteins were analyzed and displayed results as discovered by our quantitative proteomic approach: Ni^2+^-dependent regulation of danger molecule HSP70, metal-binding enolase, a fragment of skin-related BPAG protein, and albumin (Figure 1c).

**Figure 2:**
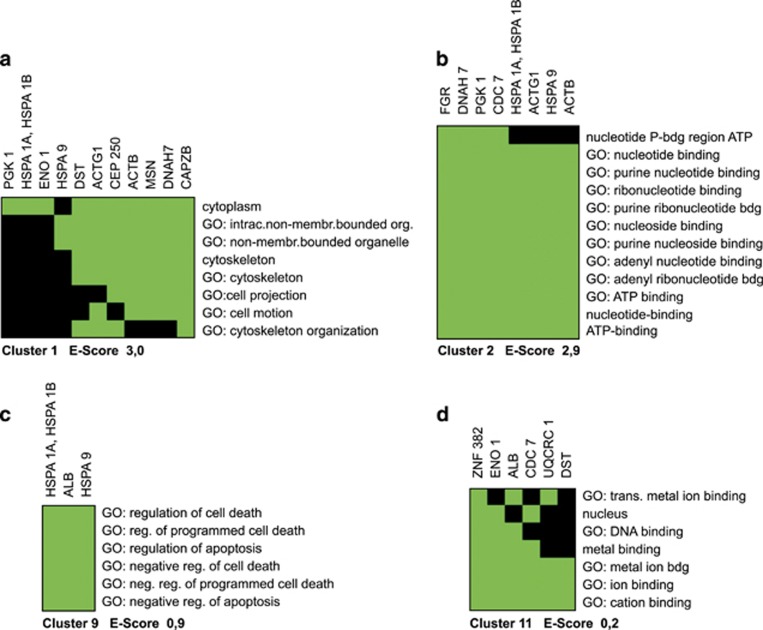
Functional annotation clustering. All Ni^2+^-regulated proteins identified by MS were subjected to web-based annotation tool and resulted in 11 functional annotation clusters with enrichment scores (*E*-score) from 3.0 to 0.2. Each group of terms represents similar biological meaning because of sharing similar protein members. Applying modified 2D viewer, four exemplary clusters are shown, with the two highest *E*-scores and (**a** and **b**) with the two lowest *E*-scores (**c** and **d**) (gray=corresponding gene-term association positively reported; black=corresponding gene-term association not reported yet)

Bioinformatics analyses of Ni^2+^-regulated monocytic proteins revealed not only clusters of metal-binding molecules or structural proteins belonging to the cytoskeleton but also metabolic enzymes and stress proteins related to hypoxia and mitochondrial function. To create useful relationship information, our data set was subjected to web-based functional annotation tool DAVID, generating relevant biological information. Eleven functional annotation clusters were generated covering over 40 annotation categories, including GO terms, PIR keywords, protein–protein interactions, protein functional domains, disease associations, biopathways, homologies, gene functional summaries, gene tissue expressions, and literature.^44^ Overall enrichment score (*E*-score), which is based on the EASE score (one-tailed Fisher's exact probability value) of each term members, ranged from 3.0 to 0.2 for all 11 clusters. Four examples are depicted (Figure 2): clusters with the two highest *E*-scores (Figures 2a and b) – top-ranked annotation groups – and two with lower *E*-scores (Figures 2c and d). Annotation cluster 1, consisting of 11 proteins, demonstrated a clear relationship to non-membrane-bounded organelles and the cytoskeleton, being further related to cellular motility (Figure 2a). Annotation cluster 2, comprising eight Ni^2+^-regulated proteins, was associated with nucleotide metabolism and ATP binding (Figure 2b). Cluster 9 was functionally linked to GO terms of (negative) regulation of cell death, apoptosis, and programmed cell death (Figure 2c), and cluster 11 was linked to metal ion binding (Figure 2d).

### Ni^2+^-induced cell death in human monocytes is independent of caspase-3/7 activity but influenced by the presence of albumin

**Figure 3:**
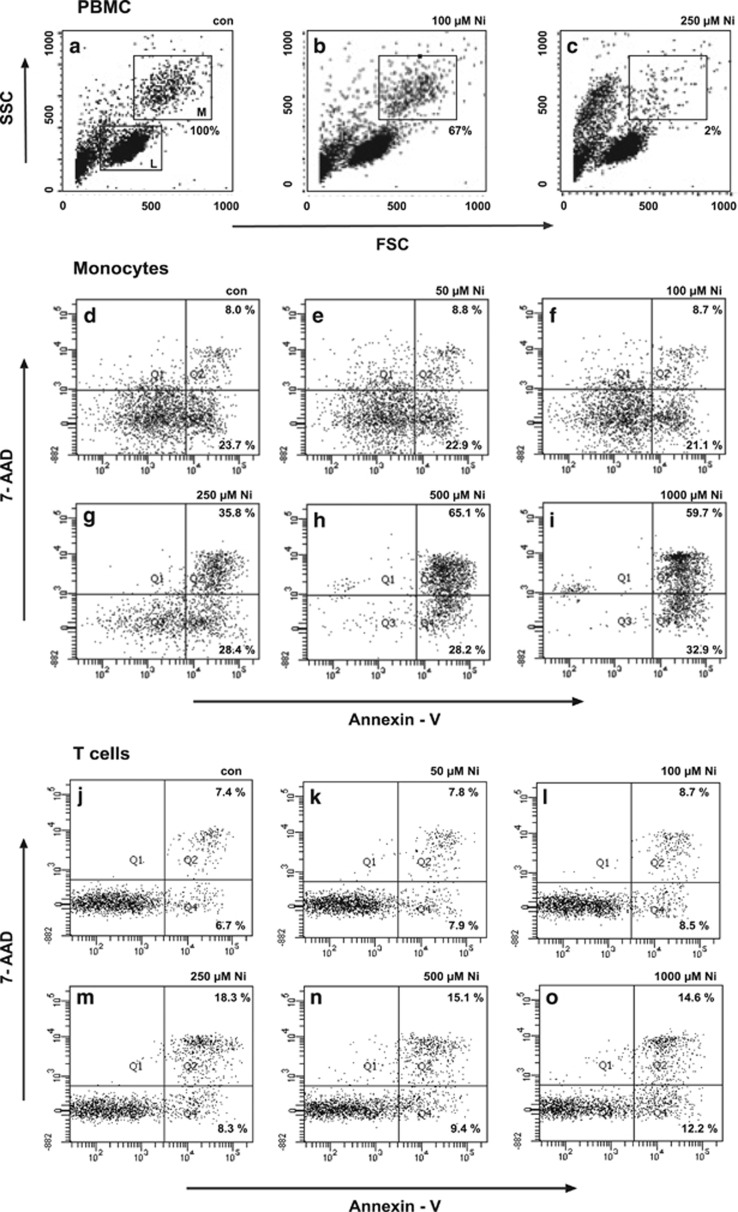
Ni^2+^-induced cell death of human monocytes. FACS analyses of PBMC (**a**–**c**), showing known lymphocyte (**l**) and monocyte populations (**m**), treated with Ni^2+^, clearly display that 98% of human monocytes are affected by treatment with 250 *μ*M NiSO_4_ (*n*=4). Cell death analyses with purified monocytes revealed Ni^2+^ concentration-dependent apoptotic monocytic cell death after incubation with 250, 500, and 1000 *μ*M NiSO_4_ (shift of cell populations to lower right and upper right dot blot quadrant; **d**–**i**). At lower Ni^2+^ concentrations in monocytes (**d**–**f**) and generally in all studies performed with purified human T cells (**j**–**o**), no significant cellular differences were measured if compared with the control. Representative plots are from three to four independent experiments. FSC, forward side scatter; SSC, side scatter; 7-AAD, 7-aminoactinomycin D

Since proteomics data were potentially linked to Ni^2+^-regulated monocytic cell death, we investigated multiple molecular processes and pathways involved in cell death by FACS analyses, cell viability assays, caspase activity determinations, and confocal microscopy. PBMCs were therefore incubated overnight (16 h) with increasing concentrations of Ni^2+^ and examined by FACS analysis. Untreated and unstained PBMC demonstrated two principal known distinct populations in the FACS dot plot (SSC/FSC) corresponding to lymphocytes (Figure 3a, con, rectangle, lower left, L) and monocytes (Figure 3a, con, rectangle, upper right, M).^45^ Significant changes were observed after incubation of cells with 250 *μ*M NiSO_4_ (Figure 3c, 250 *μ*M Ni^2+^). After Ni^2+^ treatment >90% of cells disappeared from the known monocyte region, whereas a new FACS population of cells (potentially dying cells) was detected. However, the lymphocyte fraction seemed to be unaffected (Figure 3c, 250 *μ*M Ni^2+^), and only minor changes were observed after incubation of monocytes with 100 *μ*M NiSO_4_ (Figure 3b, 100 *μ*M Ni^2+^).

Subsequent analysis to detect potential Ni^2+^-induced apoptosis by Annexin-V and 7-AAD staining revealed increasing numbers of dead Annexin-V^+^ and 7-AAD^+^ monocytes (upper right quadrant; late apoptotic/necrotic cells, Figures 3d–i) and higher numbers of Annexin-V^+^ and 7-AAD^−^early apoptotic cells (lower right; Figures 3d–i) after stimulation of isolated cells with higher concentrations of NiSO_4_ (250, 500, and 1000 *μ*M), whereas no increase in dying cells were observed after incubation with 50 or 100 *μ*M NiSO_4_. Moreover, T lymphocytes (Figures 3j–o) and B lymphocytes (data not shown) coincubated with monocytes remained less affected after the addition of even higher Ni^2+^ concentrations, with ~15% early and late apoptotic cells in untreated cells and 25% Annexin-V^+^ cells if treated with higher concentrations of NiSO_4_.

**Figure 4:**
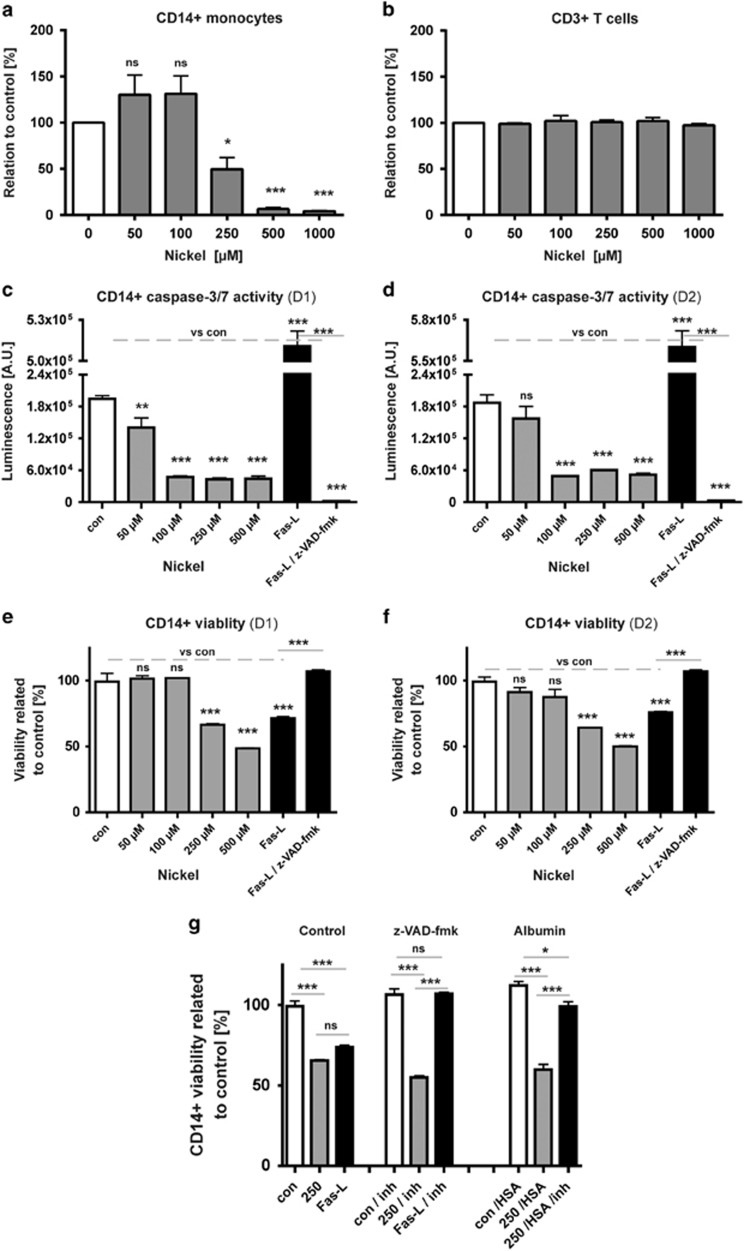
Ni^2+^ effects on caspase-3/7 activity and cell viability relative to control (**a**–**g**). Caspase-3/7 activity analyses of cells of two independent PBMC donors (D1, D2; **c** and **d**) revealed a Ni^2+^-dependent decrease in caspase-3/7 activity and differential cytotoxic effects on CD14^+^ monocytes (**e** and **f**), if compared with control and FasL induction (**c**–**f**). Incubation of human monocytes with high Ni^2+^ concentration and additional HSA with caspase inhibitor z-VAD-fmk indicates a switch from caspase-independent to -dependent cell death and subsequent cell protection (two donors; triplicates; **g**). Significant difference *P<0.05, **P<0.01 ***P<0.001. a.u., arbitrary unit; NS, nonsignificant

To determine whether observed Ni^2+^-dependent monocytic cell death is related to caspase-3 and -7 enzyme activity as in other systems, we measured their activity during NiSO_4_ treatment in combination with cell viability and cell numbers (Figures 4a–g). With increasing concentrations of NiSO_4_, we found caspase-3/7-independent monocytic cell death (>50% dead, at 500 *μ*M; Figures 4e and f), accompanied by decreased caspase-3/7 activity (Figures 4c and d). However, cells treated with FasL – a well-characterized inducer of apoptosis used by us before^46^ – induced caspase-sensitive apoptosis and therefore served as control for activity and viability.

Since human serum albumin (HSA) concentrations varied between NiSO_4_-treated monocytes and -untreated cells (Figures 1 and c and Table 1), and because the molecule is a known target for Ni^2+^ interaction, which turns it into a functional Ni^2+^-specific T-cell-activating agent,^17^ we decided to test its influence on Ni^2+^-induced monocytic cell death (Figure 4g). Remarkably, we found that incubation of cells with both HSA and caspase inhibitor during Ni^2+^ stimulation indicated a shifted caspase-3/7-dependent apoptotic monocyte cell death, which was prevented by z-VAD-fmk (Figure 4g).

### Ni^2+^-induced monocyte cell death visualized by confocal scanning microscopy

**Figure 5:**
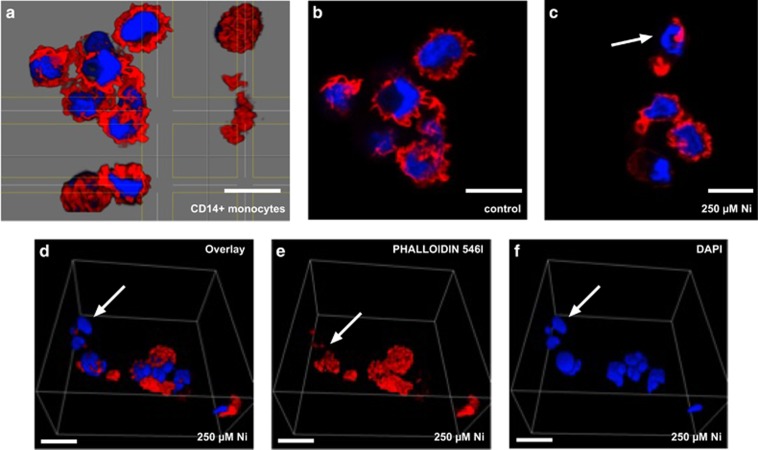
Confocal scanning microscopy analysis of Ni^2+^-induced apoptotic cell death in human monocytes. Control and Ni^2+^-stimulated cells (250 *μ*M) were stained by Phalloidin Alexa Fluor 546 (Phalloidin 546) and DAPI (**a**–**f**). Confocal z-stack images from Phalloidin- and DAPI-stained monocyte sections (**a**–**c**) demonstrated direct Ni^2+^ effect on cytoskeleton (**b**–**e**), if compared with control (**a**). Apoptosis was induced by 250 *μ*M NiSO_4_ as shown by 3D images (Imaris software) of chromatin condensation and segregation of the nucleus (**c**, **d**, and **f**)

Phalloidin Alexa Fluor 546 and DAPI were used to bind and stain cytoskeletal F-actin and nuclear DNA, respectively (Figure 5). Confocal z-stack images from phalloidin- and DAPI-stained control monocyte sections demonstrated overall cellular integrity (Figures 5a and b), whereas image overlays (z-stack or 3D) from cells treated with 250 *μ*M NiSO_4_ clearly showed cytoskeletal disruption (Figures 5c–e) and DNA condensation (Figures 5d and f), characteristic features of apoptosis.^47, 48^ Thus, data indicate Ni^2+^-specific apoptotic cell death in human monocytes.

## Discussion

In this study, we have examined Ni^2+^-dependent protein expression and functional cell death effects of the most common human contact sensitizer, nickel (Ni^2+^), in human monocytes. These cells are known precursors of phenotypically divergent DCs, organ-specific tissue macrophages, and inflammatory fibrocytes.^37, 38^ As blood-circulating, non-proliferating immune effector cells, they are able to phagocyte fragments of dead cells and process toxic and/or allergenic reactive small molecules. Thus, CD14^+^ monocytes do contribute to pathophysiological mechanisms in ACD.^49, 50, 51, 52^ Upon activation cells may produce cytokines like TNF or other factors, such as ROS or nitric oxide (NO), thereby cotriggering inflammatory processes.^53, 54, 55, 56, 57^ In addition, monocytes are capable of efficiently presenting Ni^2+^-specific epitopes to Ni^2+^-reactive human T cells^17, 58^ and producing anti-microbial factors that may be directly affected by Ni^2+^.^59^ Both Ni-containing nanoparticles and Ni^2+^ solutions have been demonstrated to influence intracellular pathways in monocytic cells such as NF-*κ*B activity, or p38 MAPK regulation, or (pro) matrix metalloproteinases 2 and 9.^30, 33, 34, 60^ Similar Ni^2+^-specific molecular effects have been described for other human cell types, such as endothelial cells,^61^ DCs^28^ and T cells,^23^ whereby Ni^2+^-related NF-*κ*B activity may be at least partially explained by direct TLR4 activation.^26^

By using modern proteomic technologies (2D-DIGE, Delta2D, MALDI-MS), we discovered 56 monocytic protein species that were significantly modulated by Ni^2+^ (Figure 1). Out of the 56 differentially regulated protein species, 22 were identified by MALDI-MS and bioinformatics analyses (Table 1). However, it has to be mentioned that for technical reasons not all Ni^2+^-regulated proteins were depicted by this kind of proteomic approach. For example, membrane proteins are usually not appropriately detected by this method. One explanation for this underrepresentation of membrane proteins by 2-DE is that they are generally not highly abundant in whole-cell lysates, and that many of them possess more alkaline isoelectric points so that they are not visible in our standard 2D gels. However, the main reason for the lack of membrane proteins is that many of them are poorly soluble in aqueous buffers used and necessary in 2D electrophoresis. In contrast to cytosolic proteins, these proteins are usually hydrophobic and generated to be soluble in lipid bilayers and not in water or buffers for isoelectric focusing (IEF) (first dimension).^62, 63^ Since annotation analyses of candidate proteins revealed Ni^2+^-related molecular processes of cell death, cytoskeleton organization, and metal ion binding, additional functional assays of cell death and caspase activity were performed.

Caspase-dependent apoptosis is regarded as one homeostatic death program controlling numerous (patho)physiological processes including the numbers of circulating immune cells in human blood like short-lived monocytes.^64, 65^ However, distinct subsets of monocytes seem to be more affected in undergoing spontaneous caspase-dependent apoptosis than others.^66^ In addition, alternative capase-independent cell death pathways do exist, which may be characterized by DNA fragmentation and partial chromatin condensation that are similar to our observations (Figure 5).^67, 68^ Other types of leukocyte cell death reveal another distinct form of apoptosis,^69^ being positive for Annexin-V staining and mitochondrial membrane potential disruption, but being not accompanied by cytochrome *c* or AIF release and also independent of caspase-3 activation, as it has been observed by us for Ni^2+^-treated monocytes (Figure 4). On the other hand, if human monocytes are treated for 24 h with 2-chlorodeoxyadenosine, an immunosuppressive drug, caspase-3- and -9-dependent apoptosis has been demonstrated, but not under the same conditions for monocyte-derived DCs, indicating distinct cell-type- and chemical-specific apoptotic mechanisms.^70^ This view is further supported by other nickel-related studies.^71, 72^ Furthermore, even though Ni^2+^ treatment may affect TLR4 pathway activation and thereby may trigger another specific type of cell death, called necroptosis, there was no typical sign of intact nuclei and uncontrolled accidental necrosis, thus likely excluding this type of cell death in human monocytes.^73^

HSA represents another metal-binding molecule identified in this study (Figure 1 and Table 1). Besides many other molecular functions HSA is known to bind Ni^2+^ and Cu^2+^ more specifically and more tightly than other metal cations. However, HSA is not only a multifunctional shuttling molecule and detoxifier in human blood but further may act immunologically when complexed to Ni^2+^ ions to mediate Ni^2+^-specific human T-cell activation.^3,17^ Moreover, albumin may inhibit – as a possible negative regulator of cell death (Figure 2) – induced processes of cell death and apoptosis,^74, 75^ thus possibly explaining higher concentrations of HSA observed after applying high concentrations of Ni^2+^ to human monocytes (Figure 1). However, in other cell types like human tubular cells albumin may have reverse effects.^76^ No protective effect was found in our study in the presence of additional HSA alone during Ni^2+^-specific stimulation, but a significant protective effect was observed by applying HSA in combination with caspase inhibitor z-VAD-fmk, thus suggesting a possible shift from caspase-insensitive Ni^2+^-specific cell death to a caspase-dependent Ni^2+^-specific cell death (Figure 4g).

Depending on the model used Ni^2+^-specific (patho)physiological regulation may be distinctly influenced by the concentration of Ni^2+^ applied. For example, in contrast to Ni^2+^-dependent monocytic cell death observed here, Schmidt *et al.*,^26^ reported treatment of human monocytes, at 4–6-fold higher concentrations of Ni^2+^ (1–1.5 mM; MA Freudenberg, personal communication) to study activation of TLR4, but without presenting detailed information on monocytic cell viability after metal treatment.^26^ Similar Ni^2+^ concentrations were applied in experimental settings with primary human umbilical vein endothelial cells and keratinocytes,^71^ whereas lower concentrations were used by Antonios *et al.*^30^ to investigate Ni^2+^-dependent mechanisms of IL-12 synthesis in DC. Some Ni^2+^-specific T clones are already activated at concentrations of 100 *μ*M Ni^2+^ or even lower,^17^ whereas Ni^2+^-specific ear swelling in mice is induced with a 100-fold higher concentration of Ni^2+^. This raises the principal question of how to translate experimental animal data to human disease phenotypes. In addition, T cells treated under the same conditions as monocytes demonstrated that human T-cell viability seemed to be significantly less affected by Ni^2+^ compared with human monocytes (Figures 3a–c and Figures 4a and b).

Another identified Ni^2+^-regulated protein that may influence the metal-specific cellular response included heat-shock protein 70 (Hsp70). Danger molecule Hsp70 – or related Hsp70 family members – represented one type of Ni^2+^-regulated protein identified in our study (Figure 1 and Table 1) clustering to both regulation of cell death and to nucleotide/ATP binding (Figure 2 and Table 1).^21^ Stress protein Hsp70 is known to be affected by metal ion binding (Mg^2+^) – and possibly also by Ni^2+^ ion binding.^9^ Moreover, depending on the type of inducers of immunogenic cell death in cancer cells, Hsp70 does have a role in distinct associated DAMPs (damage-associated molecular patterns), thereby being also associated with several stages of apoptosis (early/mid and mid/late apoptotic stages), a view that might have additional implications for nickel-induced monocyte cell death.^77^

In summary, our data strongly suggest Ni^2+^-dependent monocytic cell death before Ni^2+^-specific T-cell activation in human skin hypersensitivity at concentrations of Ni^2+^ higher than 250 *μ*M. Data clearly demonstrate Ni^2+^-dependent monocytic cell death independent of caspase-3/7 activity, but exhibiting cellular characteristics of apoptotic cell death. In contrast to monocytes, human T cells seem to be less affected if treated with the same concentrations of Ni^2+^. However, as both cell types, T cells and monocytes, or monocyte-derived DCs are regarded to (co)trigger Ni^2+^-specific T-cell activation in human nickel allergy, it is tempting to suggest that in human skin under pathophysiological conditions monocyte contact with Ni^2+^ concentrations above 250 *μ*M may induce inflammatory cell death reactions, whereas contact with Ni^2+^ concentrations below 250 *μ*M may contribute to adaptive type IV immune responses.^58, 78^ Thus, elucidation of all Ni^2+^-triggered effects in human immune cells will give a better molecular and cellular understanding of human ACD and may also support the development of novel therapeutic intervention.

## Materials and Methods

### Isolation of human monocytes

Human buffy coats were generated at the Institute of Transfusion Medicine and Immunology, University of Heidelberg, Medical Faculty of Mannheim, Germany. PBMCs from buffy coats were isolated by Ficoll-Hypaque density gradient centrifugation (Biocoll-Separation-Solution, density 1.077 g/ml; Biochrom AG, Berlin, Germany), and monocytes positively sorted by applying MACS sorting technique with CD14 Micro Beads (isotype mouse IgG2a; Miltenyi Biotec GmbH, Bergisch-Gladbach, Germany) and QuadroMACS Separator on LS-columns (Miltenyi Biotec GmbH) according to the manufacturer's guidelines. Successive FACS analysis demonstrated a purity of >90% monocytes in each selected sample (*n*=4). Parallel experiments were performed with PBMC cultures, and monocytes and T cells were analyzed separatively for each sub-population. The study was approved by the ethics committee of the University of Heidelberg and all blood donors gave informed consent.

### Human monocyte stimulation

Samples of isolated monocytes were cultured in X-Vivo-10 cell medium (1 × 10^6^ cells per ml X-Vivo-10; Bio Whittaker, Verviers, Belgium) at 37 °C (CO_2_ incubator, 5% CO_2_ atmosphere) and the following concentrations of NiSO_4_ were added: 50, 100, 250, 500, or 1000 *μ*M *versus* control. After an incubation period of 16 h, cells were harvested. Cell lysis was performed as described previously.^9^ In brief, cells (2 × 10^6^/ml) were lysed by using 1 ml Triton lysis buffer (137 mM NaCl, 20 mM Tris-HCl, 0.1% Triton X-100, 10% glycerol, plus protease inhibitor cocktail (Roche Diagnostics, Mannheim, Germany); pH 8.2) for 1 h at 4 °C. Cell fragment- and nuclei-free samples were generated by centrifugation (14 000 r.p.m. for 10 min at 4 °C) and protein concentrations determined by Coomassie Plus Protein Assay Reagent following the manufacturer's instructions (Pierce, Rockford, IL, USA). Subsequently, samples were frozen at −80 °C.

### Protein labeling for 2-DE

To achieve adequate staining with amine-reactive cyanine dyes (CyDye-DIGE colors CyDye-DIGE Fluor Cy2/3/5 (minimal dye); GE Healthcare, Munich, Germany), protein samples were first adjusted to pH 8.5–9.0 by adding 2 *μ*l 30 mM Tris-HCl (pH 8.8) in a stepwise manner. Labeling was performed shortly before IEF according to the manufacturer's protocol. Before labeling 2 *μ*l of CyDye was diluted in 3 *μ*l DMF (*N*,*N*-dimethylformamide) to 400 pmol CyDye. As an internal standard, 25 *μ*g of protein (mixture of same amount of proteins from negative control, 50 and 250 *μ*M NiSO_4_ stimulation) were labeled with 200 pmol of Cy2, 25 *μ*g protein of the negative control were labeled with Cy3 and 25 *μ*g of protein from the NiSO_4_-stimulated samples (50 or 250 *μ*M, respectively) were labeled with Cy5. During labeling, the samples were mixed, incubated for 30 min in the dark on ice, and the reaction was stopped by adding 1 *μ*l of 10 *μ*M lysine. Differentially labeled samples were pooled and transferred to IEF. From each of the four donor samples four independent experiments were performed.

### Protein separation

2D electrophoresis was carried out essentially as reported.^9^ After rehydration of IPG strips (6 M urea, 2 M thiourea, 2% CHAPS, bromphenol blue, 32 mM DTT, 1% IPG buffer for either pH 3–10 NL or pH 4–7 linear), each strip was loaded analytically with 75 *μ*g CyDye-labeled protein mixture (in-gel rehydration, overnight), including technical replicates. IEF was performed at 20 °C by using Ettan IPGphor (GE Healthcare).

After equilibration (6 M urea, 30% glycerol, 2% SDS, 50 mM Tris (pH 8.80), bromphenol blue, in aqua dest., 20 min), IEF strips were reduced with 1% DTT, and then alkylated with 2.5% iodoacetamide at RT (20 min). They were then transferred to SDS gels (12.5% SDS, 30% acrylamide/bisacrylamide, 1.5 mM Tris (pH 8.8), 10% APS, 0.03% TEMED; 200 × 250 × 1.5 mm^3^ spacer) in low-fluorescence glass plates (GE Healthcare). Second dimension was performed overnight at 20 °C (4 h at 40 V, following 130 V for 16 h) with an Ettan DALT Large Vertical System (GE Healthcare).

To evaluate the running behavior of the monocyte proteins in 2-DE, 2-DE tests were performed before starting the DIGE experiments. Therefore, from four selected protein samples (negative controls and stimulated samples), an amount of 20 *μ*g protein was separated from each sample in the first dimension with IPG strips pH range 3–10 and pH range 4–7 (Ready Strips IPG Strips; 24 cm, pH 3–10 nonlinear/pH 4–7 linear; Bio-Rad Laboratories, Hercules, CA, USA) as described above without DIGE labeling. Second dimension was also performed as described above. Each assay was performed twice with a technical replicate to achieve concordant results. After protein fixation (2 h, 40% ethanol, 10% acetic acid), the gels were stained for 3 h with flamingo pink solution (Flamingo Pink; Bio-Rad Laboratories). Background staining was reduced by incubation with 0.1% Tween (10 min) and gels were scanned in a Fujifilm FLA 5100 laser scanning system (437 nm laser, 100 *μ*m pixel size resolution, 600 V; Fujifilm, Düsseldorf, Germany). Images were generated with Multi Gauge software (V3.0; Fujifilm).

### Protein visualization

After finishing second dimension, DIGE gels were directly transferred to a FLA 5100 scanning system (Fujifilm) and scanned twice inside their low-fluorescence glass plates with laser and filter settings appropriate for CyDyes according to the supplier's protocol (GE Healthcare). Image contrast was displayed with application of Multi Gauge software (V3.0; Fujifilm) and scanned gels were cooled (4 °C) until spot picking process and protein species identification was executed.^42, 43^ Statistical analyses of stained or labeled samples were performed using the Delta2D software (Decodon, Greifswald, Germany).

### Image analysis of differential protein regulation

Differential gel analysis was performed by applying Delta2D software from Decodon. Spot matching was manually coedited by in-gel standard warping strategy in each of the 12 DIGE images. Primarily the internal standard protein spots were displayed as green spots, the spots containing the negative control or stimulated protein mixture subsequently in a red color. Matching resulted in either green spots, when the protein amount of the negative control was higher, or a red spot for higher protein concentration in the stimulated probe. Yellow spots resulted when the same fluorescence amount – same protein concentration in both samples–was detected. For correct identification, and according to Delta2D software protocol, a fusion image of the 4 × 3 digitalized gels was produced of regulated spots (average size of spots 25 pixel, sensitivity 90%) and the detected spots were transferred to all the images (4 gels per lysate, CyDye 2/3/5 image each). Regulation factors for differentially regulated proteins were set: for upregulated proteins >1.5, and for downregulated proteins <0.66, with unregulated protein spots ranging from 0.66 to 1.5. Not every regulated spot detected on the fusion image could be found on every single DIGE image, but if protein regulation was detected on several corresponding gel replicates, spots were handled as potential candidates for Ni^2+^-regulated proteins and included for MALDI-MS analysis.

### Preparative gels, staining, and trypsinization for protein identification by MS

To gain protein concentrations sufficient for MALDI-MS analysis, preparative gels with a mixture from all buffy coat samples were generated, two gels with 740 *μ*g protein per gel, and two gels with 387 *μ*g protein per gel. To achieve a higher protein concentration, mixed samples were precipitated by standard acetone precipitation. In a final step, acetone was removed and protein pellets dried by air. Protein concentrations were determined by Bradford analysis, and 2-DE run as described before. After protein fixation (40% ethanol, 10% acetic acid, 2 h), preparative gels were stained with flamingo pink solution as described above and scanned for protein spot visualization, followed by mass spectrometric compatible silver staining. Stained protein spots were excised manually and stored overnight (−20 °C). Then, trypsin stock solution (Promega, Fitchburg, WI, USA) was diluted 1 : 50 with 40 mM NH_4_HCO_3_ and a washing step followed before proteins were reduced and alkylated: (1) 100 *μ*l aqua dest., 5 min, 42 °C, 600 r.p.m.; (2) 100 *μ*l 40 mM NH_4_HCO_3_/ethanol 50 : 50, 5 min, 42 °C, 600 r.p.m.; (3) 100 *μ*l 10 mM DTT, 1 h, 56 °C, 600 r.p.m.; (4) 100 *μ*l 40 mM NH_4_HCO_3_, 5 min, 42 °C, 600 r.p.m.; (5) 100 *μ*l iodoacetamide, 30 min, 25 °C, 600 r.p.m.; (6) 3 × 150 ml 40 mM NH_4_HCO_3_ and 100% ethanol alternately for 8 min, 37 °C, 600 r.p.m. Dehydration of protein spots was performed by repeated addition of 100 *μ*l 100% acetonitrile (ACN, 1 min, 25 °C, 600 r.p.m.) and air-drying (15 min). For protein trypsinization, 5 *μ*l of trypsin (1:50) was added, samples centrifuged (500 r.p.m., 30 min, 25 °C) and gel pieces covered with 40 mM NH_4_HCO_3_ (37 °C, overnight), before being stored at −20 °C until MS analysis.

### Protein identification by MS and western blotting

Dried trypsinized samples were dehydrated (3x, 0.1% TFA/ACN (v/v 50 : 50)), washed, and equilibrated with 0.1% TFA. Peptides were purified and enriched by using C18 ZipTip columns according to the supplier's protocol (Millipore, Bedford, MA, USA). Peptide samples were concentrated by vacuum centrifugation (Savant AES 1010; GMI Incorporation, Ramsey, MN, USA) and again diluted in 0.1% TFA. Peptides were then diluted 1 : 7 in HCCA (*α*-cyano-4-hydroxy-cinnamic acid; in ethanol, 0.1%TFA solution) and spotted on the anchor chip target plate. Mass spectrometry for protein identification was performed automatically using Bruker Ultraflex II (nitrous oxide laser, 337 nm wavelength, pulse duration 4 ns, irradiated area 50–100 *μ*m; Bruker Daltonics, Bremen, Germany). The mass spectrum of peptide fragments ranged from 700 to 3500 *m*/*z*. External calibration was carried out periodically using a peptide standard (Peptide Calibrant 2; Bruker Daltonics). By using MASCOT software (SwissProt databank), protein search took place with the following limitations: human proteins (*Homo sapiens*), only one missed cleavage per protein and the possibility of oxidation at methionine. Mass tolerance was set to ±100 p.p.m. and identified proteins had to have a statistically significant Mowse score of at least 56.

Ni^2+^-dependent regulation of monocytic proteins was confirmed by complementary western blotting. BPAG and enolase antibodies were purchased from Santa Cruz Biotechnology (Heidelberg, Germany), and Hsp70 antibody from Stressgen (Vancouver, BC, Canada).

### FACS cell death analysis

PBMCs were isolated from buffy coats by Ficoll-Hypaque density gradient centrifugation as described above. FACS analysis was performed as described.^17, 45^ For examination of apoptosis, monocytes were washed with PBS, and then stained for Annexin-V using FITC and with propidium iodide or 7-AAD according to the manufacturer's protocol (BD Pharmingen, Heidelberg, Germany), and analyzed by flow cytometry.^79^

### Caspase activity and viability assay

To measure monocytic capase-3 and -7 activity and to distinguish between caspase-dependent and -independent cell death, cells were incubated with NiSO_4_ salt solutions as indicated and caspase-3 and -7 activity examined by using the Caspase-Glo 3/7 Assay (Promega, Fitchburg, WI, USA). Caspase inhibitor z-VAD-fmk was purchased from Bachem (Weil, Germany). Cell viability was measured by WST-1 assay according to the manufacturer's protocol (Roche Diagnostics, Mannheim, Germany).

### Apoptosis measurement by confocal microscopy

For fluorescent detection of cell death and apoptosis by confocal microscopy, cells were treated with NiSO_4_ as described before and labeled with Phalloidin Alexa Fluor 546 for cytoskeletal F-actin (Molecular Probes, Invitrogen, Mo Bi Tec GmbH, Goettingen, Germany) and DAPI (diamidino-2-phenylindole) as classical nuclear counterstain (Mayeur, FEBS 2002). Cells were washed, fixed in 2% paraformaldehyde (15 min; 4 °C; dark), and embedded carefully in Fluoromount-G (Southern Biotechnology Associates, Birmingham, AL, USA), before imaging by confocal microscopy with the TCS SP2 UV System (Leica Microsystems, Mannheim, Germany). Imaris software was used for all analyses of images (Bitplane, Zurich, Switzerland). In some cases, stacks were rendered into 3D volumes. At least 10 different, random microscopic fields (>100 cells per field) were analyzed for each sample.

### Statistics

Experiments were performed with human blood samples (*n*=3–5), plus their technical replicates. This resulted in 16 different 2D-DIGE gels with three different laser DIGE images (Cy2/3/5) each. Single protein spot detection and regulation was performed by applying statistically significant Delta2D analyses (Student's *t*-test, differences at *P*<0.05 were considered as statistically significant (Decodon Delta2D software; Decodon). Viability and caspase data were analyzed by using the GraphPad Prism software 6.05 (GraphPad Software Inc., La Jolla, CA, USA).

## Figures and Tables

**Table 1:** Mass spectrometric identification of Ni-regulated proteins in human monocytes

**No.**	**Protein**	**Accession number**^a^	**Regulation**	**Sequence coverage (%)**	**Peptide queries matched**	**Percental peptide matched**	**Score**^b^	**MW (Da)**	**pI**
1	Bullous pemphigoid antigen 1, isoforms 1/2/3/4/5/8 (fragment)	sp|Q03001|BPA1_HUMAN	−	10	32	48.5	74	374 544.0	6.38
2	Centrosome-associated protein CEP250	sp|Q9BV73|CP250_HUMAN	−	25	12	18.2	60	281 880.0	5.00
3	Actin, cytoplasmic 1	sp|P60709|ACTB_HUMAN	−	30	9	13.6	56	42 052.0	5.29
4	Actin, cytoplasmic 2	sp|P63261|ACTG_HUMAN	−	30	9	13.6	56	42 108.0	5.29
5	Proto-oncogene tyrosine-protein kinase FGR	sp|P09769|FGR_HUMAN	−	23	13	14.8	58	60 068.0	5.4
6	Alpha-enolase	sp|P06733|ENOA_HUMAN	−	40	17	20.0	104	47 481.0	7.01
7	Serum albumin	sp|P02768|ALBU_HUMAN	+	27	20	21.1	92	71 317.0	5.92
8	Serum albumin	sp|P02768|ALBU_HUMAN	+	25	19	27.1	108	71 317.0	5.92
9	Stress-70 protein, mitochondrial	sp|P38646|GRP75_HUMAN	−	20	11	35.5	75	73 920.0	5.87
10	Alpha-enolase	sp|P06733|ENOA_HUMAN	−	47	20	29.9	181	47 481.0	7.01
11	Actin, cytoplasmic 1	sp|P60709|ACTB_HUMAN	−	37	14	16.5	81	42 052.0	5.29
12	Actin, cytoplasmic 2	sp|P63261|ACTG_HUMAN	−	37	14	16.5	81	42 052.0	5.29
13	Dynein heavy chain 7, axonemal	sp|Q8WXX0|DYH7_HUMAN	−	11	33	38.4	59	464 382.0	5.70
14	Zinc-finger protein 382	sp|Q96SR6|ZN382_HUMAN	+	26	15	20.3	70	65 122.0	9.40
15	Serum albumin	sp|P02768|ALBU_HUMAN	+	29	19	21.8	99	71 317.0	5.92
16	Phosphoglycerate kinase 1	sp|P00558|PGK1_HUMAN	−	51	15	16.5	80	44 985.0	8.30
17	Moesin	sp|P26038|MOES_HUMAN	−	24	20	25.0	78	67 892.0	6.08
18	Cell division cycle 7-related protein kinase	sp|O00311|CDC7_HUMAN	−	26	13	17.3	59	64 646.0	8.96
19	F-actin-capping protein subunit-β	sp|P47756|CAPZB_HUMAN	+	36	12	14.0	73	31 616.0	5.36
20	Cytochrome *b*–*c*1 complex subunit 1	sp|P31930|QCR1_HUMAN	−	36	11	22.00	84	53 297.0	5.94
21	Heat-shock 70 kDa protein 1	sp|P08107|HSP71_HUMAN	−	24	12	13.8	56	70 294.0	5.48
22	Heat-shock 70 kDa protein 1 Chain A/B	sp|P08107|HSP71_HUMAN	−	28	10	10.0	88	41 973.0	6.69

Mass spectrometric identification of Ni^2+^-regulated proteins in human monocytes. Differentially regulated proteins were set: for upregulated proteins >1.5, and for downregulated proteins <0.66, with unregulated protein species ranging from 0.66 to 1.5 (*n*=3)

a Proteins were identified using MASCOT search engines, with a given significant score

b Accession numbers are from UniProtKB/SwissProt (http://www.uniprot.org/). Regulation is given qualitatively (−=down; += up)
